# Congenital Dyserythropoietic Anemia Type I: A Rare Case Report

**DOI:** 10.7759/cureus.48594

**Published:** 2023-11-10

**Authors:** Vaidehi Nagar, Nanda J Patil

**Affiliations:** 1 Pathology, Gujarat Medical Education and Research Society (GMERS) Medical College and Hospital, Valsad, IND; 2 Pathology, Krishna Vishwa Vidyapeeth (Deemed to Be University), Karad, IND

**Keywords:** anemia, pediatric age group, hereditary disorder, congenital dyserythropoietic anemia, bone marrow aspiration

## Abstract

Congenital dyserythropoietic anemias are a group of rare hereditary conditions affecting erythropoiesis. These disorders are characterized by anemia, primarily caused by inefficient erythropoiesis, as well as distinctive morphological abnormalities observed in most erythroblasts in the bone marrow. congenital dyserythropoietic anemia type I (CDA-I) is a hereditary condition characterized by inefficient production of red blood cells and excessive accumulation of iron. It follows an autosomal recessive pattern of inheritance. There have been approximately 300 recorded cases of CDA-I documented on a global scale. CDA-I is a rarely documented condition in the Indian subcontinent. Therefore, we will be examining a case of CDA-I in the present article. A male infant, aged four months, who had signs of vomiting, weight loss, and failure to thrive, was diagnosed with CDA-I following a bone marrow aspiration. Our experience provides further evidence supporting the notion that the accurate diagnosis of CDA-I can be achieved by doing a comprehensive assessment of bone marrow aspiration.

## Introduction

Congenital dyserythropoietic anemia (CDA) is a hematological disorder that is rather rare and has been predominantly documented in Central and Western Europe as well as North Africa [1-3]. There have been approximately 300 recorded cases of congenital dyserythropoietic anemia type I (CDA-I) documented on a global scale [3,4].

CDAs refer to a collection of rare genetic disorders affecting erythropoiesis. These conditions are primarily characterized by anemia resulting from inefficient erythropoiesis as well as distinctive morphological abnormalities observed in most erythroblasts in the bone marrow. CDA-I is a genetic condition characterized by autosomal recessive inheritance, resulting in inefficient production of red blood cells and excessive accumulation of iron [3,4]. The two most prevalent types are CDA-II and CDA-III, with the nonfamilial subtype being the least common. The majority of instances of CDAs exhibit an autosomal recessive inheritance pattern [5]. It consists of four distinct categories, namely CDA-I, CDA-II, CDA-III, and CDA-IV. These classifications are determined based on the examination of bone marrow morphology, clinical characteristics, and genetic variations. Furthermore, there are also additional subgroups and variants that have been found [6].

CDA-I is rarely documented in the Indian subcontinent; hence, we will be examining a case of CDA-I. Our example additionally underscores the observation that the diagnosis of CDA-I can be established with a high degree of reliability through detailed evaluation of bone marrow aspirates. Hence, it is imperative to take all patients presenting with chronic anemia into account.

## Case presentation

A four-month-old male child was brought by his parents to the pediatric outpatient department of our institute. Parents gave a history of vomiting for one month, loss of weight, and failure to thrive. On clinical examination, the child was pale and lethargic with splenomegaly. Clinically, the child was evaluated for anemia. All routine blood investigations were done (Table 1).

**Table 1:** Laboratory investigations Hb: Hemoglobin; TLC: Total leukocyte count; PLT: Platelets; HCT: Hematocrit; MCH: Mean corpuscular hemoglobin; MCHC: Mean corpuscular hemoglobin concentration; MCV: Mean corpuscular volume; ESR: Erythrocyte sedimentation rate

Laboratory tests	Results	Normal value
Hb	5.3gm/dl	12.0-16.0gm/dl
TLC	17100/mm^3^	4000-11000/mm^3^
PLT	75000/mm^3^	1.50-4.50L/mm^3^
HCT	15.8%	35-50%
MCH	18pg/cell	26-32pg/cell
MCHC	20gm/dl	32.0-36.0gm/dl
MCV	92.8fl	80-100fl
ESR	45mm/hr	0-10mm/hr
Retic	1.8%	0.5-2%
Serum bilirubin	18mg/dl	<15mg/dl

The differential leukocyte count of the patient revealed lymphocytosis with few atypical blastoid-type cells and nucleated red blood cells (Table 2).

**Table 2:** Differential leukocyte count findings DLC: Differential leukocyte count; nRBC: Nucleated red blood cell; WBC: White blood cells

DLC	Findings	Normal value
Neutrophils	4%	45-65%
Lymphocytes	88%	25-45%
Eosinophils	1%	1-6%
Monocytes	3%	2-10%
Atypical blastoid-type cells	4%	-
nRBC	6/100 WBC	-

**Figure 1:**
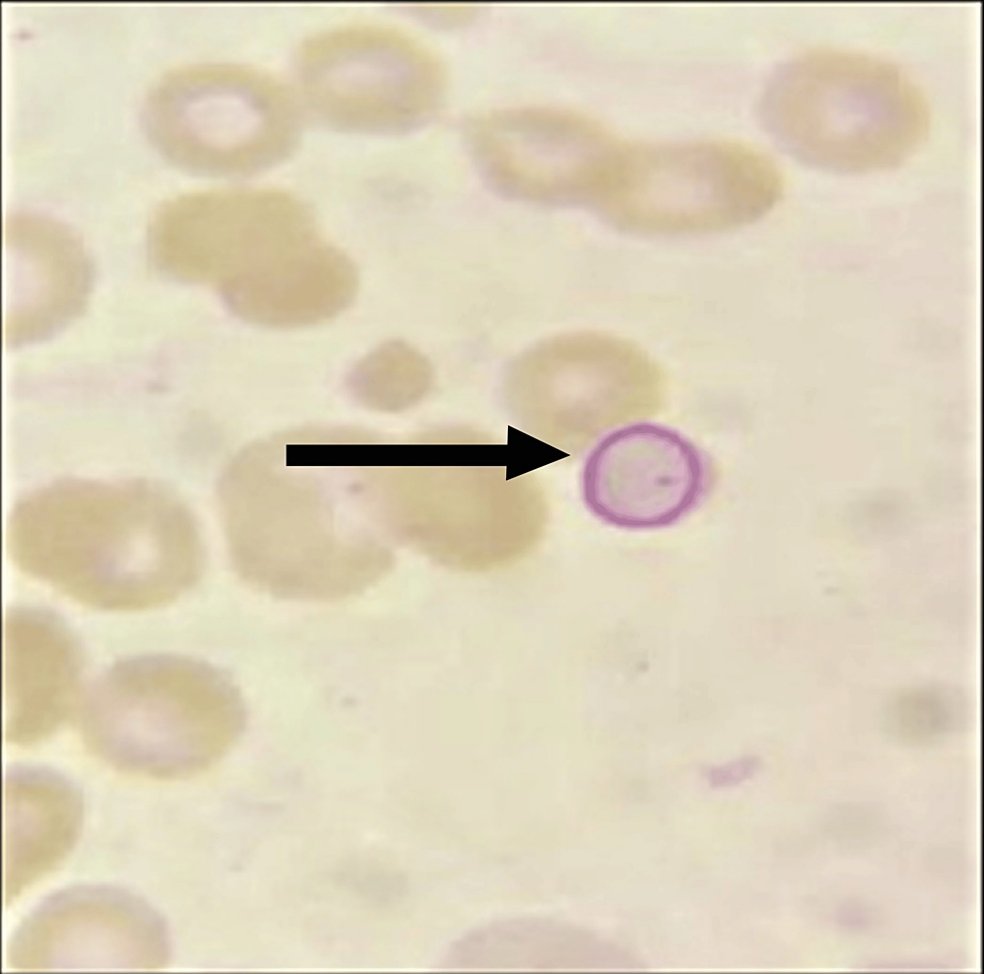
Peripheral blood smear, Leishman stain, 1000x magnification The Cabot ring is denoted by a black arrow

**Figure 2:**
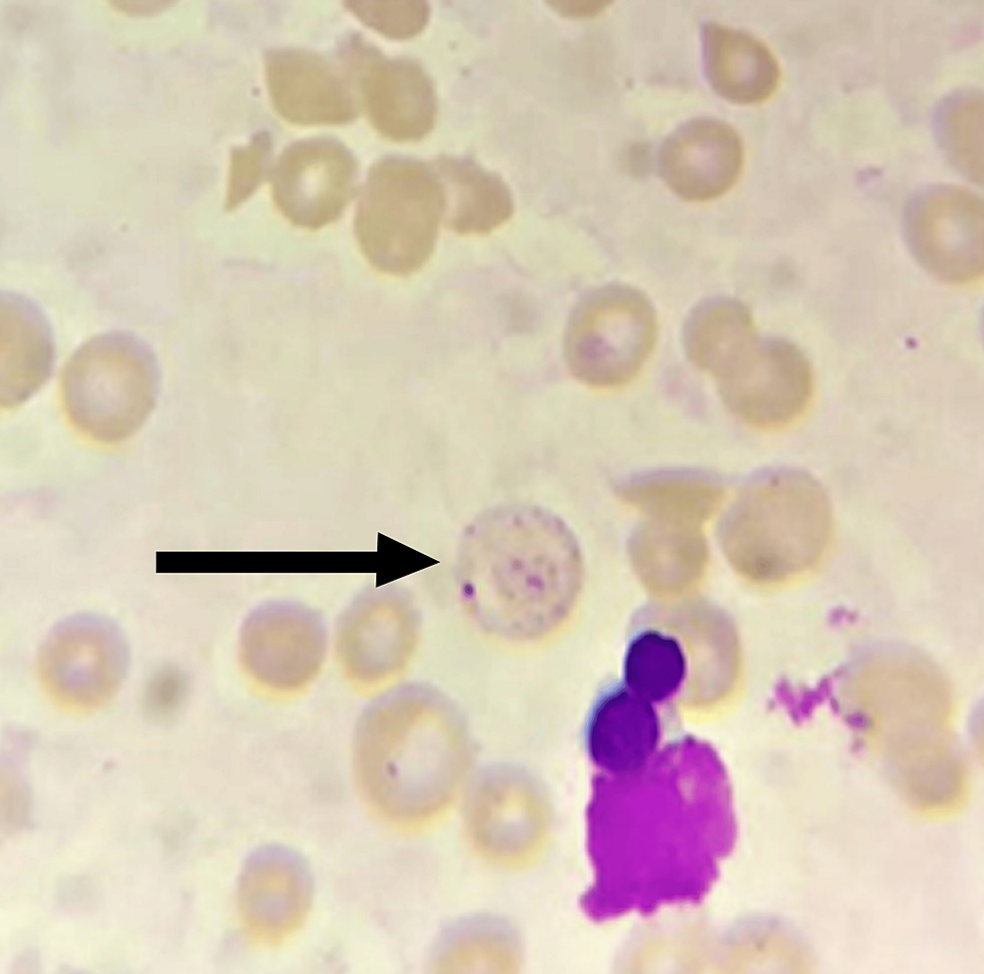
Peripheral blood smear, Leishman stain, 1000x magnification Basophilic stippling is denoted by a black arrow

**Figure 3:**
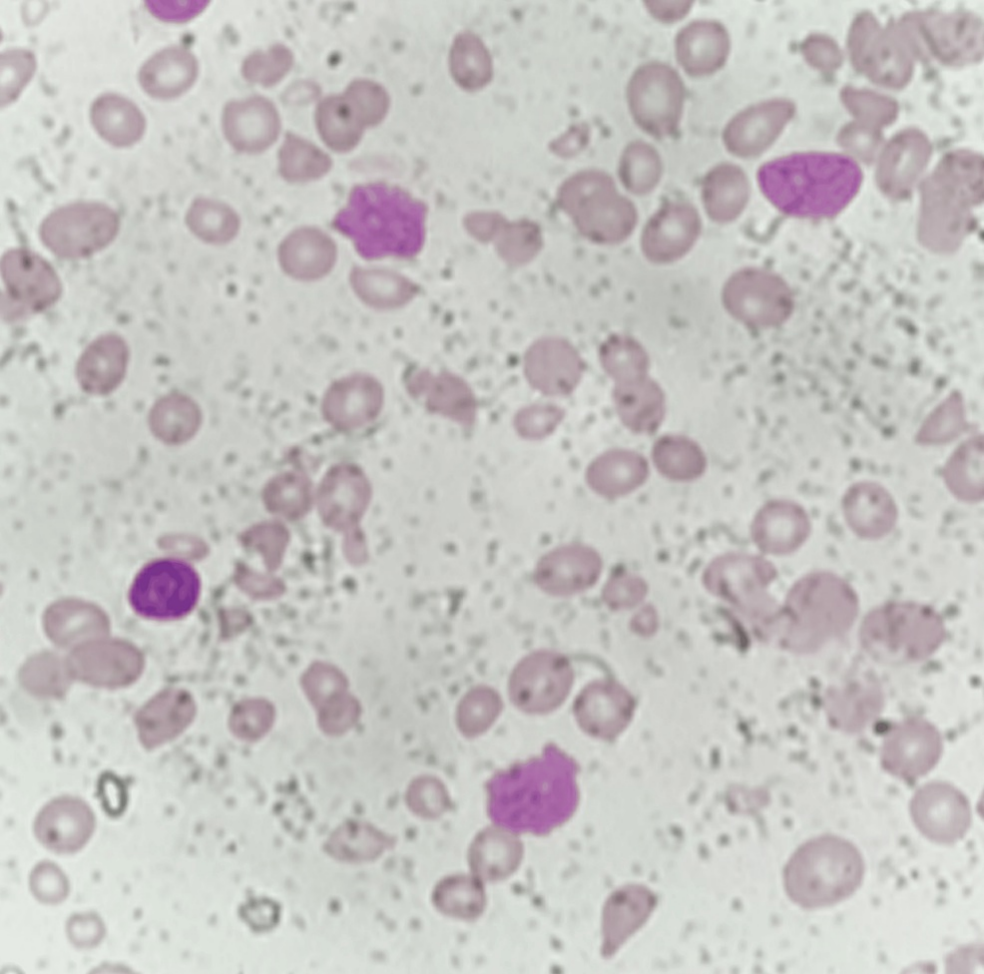
Peripheral blood smear, Leishman stain, 400x magnification Entire smear shows dyserythropoietic changes in red blood cells

**Figure 4:**
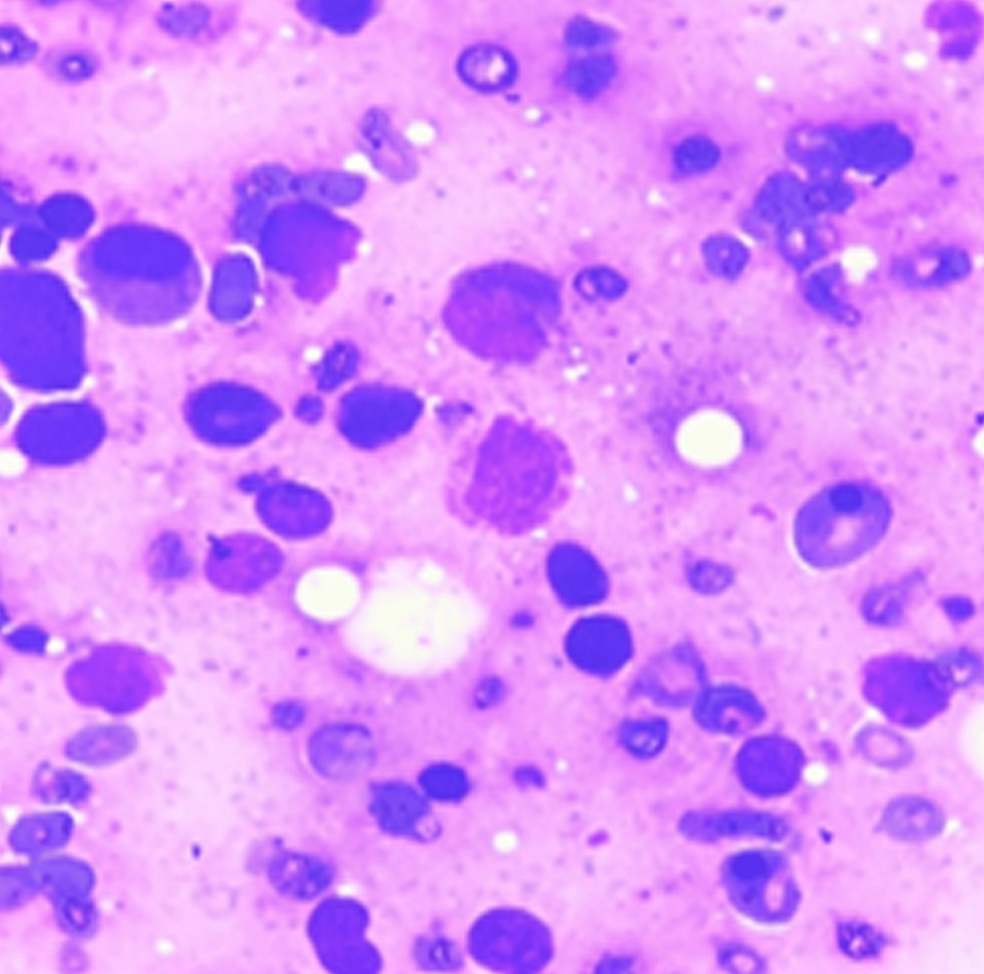
Peripheral blood smear, Leishman stain, 400x magnification The entire picture shows dyserythropoietic changes

A peripheral blood smear revealed dimorphic anemia (Table 3) (Figures 1-4).

**Table 3:** Features noted in peripheral blood smear RBC: Red blood cells

Peripheral blood smear	Findings
Red blood cells	Anisopoikilocytosis ++ to +++, macrocytes++, microcytes +++ with ovalocytes, teardrop, pencil cells, target cells, fragmented RBC, basophilic stippling, Cabot ring and nucleated RBC noted
White blood cells	Lymphocytosis with reactive changes seen
Platelets	Thrombocytopenia
Parasites	Nil
Diagnosis	Dimorphic anemia

Bone marrow aspiration findings revealed CDA-I (Table 4).

**Table 4:** Bone marrow aspiration findings CDA: Congenital dyserythropoietic anemia

Bone marrow aspiration	Findings
Predominant cells	Erythroid series
Erythroid series	Normoblastic and megaloblastic erythropoiesis. Dyserythropoietic features including multinucleation and budding of the nucleus also seen.
Granulocytic series	All forms were seen; many smudge cells seen
Myeloid/erythroid ratio	2:1
Lymphocytes	Few, mature
Monocytes	Few, mature
Plasma cells	Few, mature
Megakaryocytes	Suppressed, few with dysplastic features
Abnormal cells and parasite	Nil
Bone marrow iron	Increased
Diagnosis	CDA Type I

**Figure 5:**
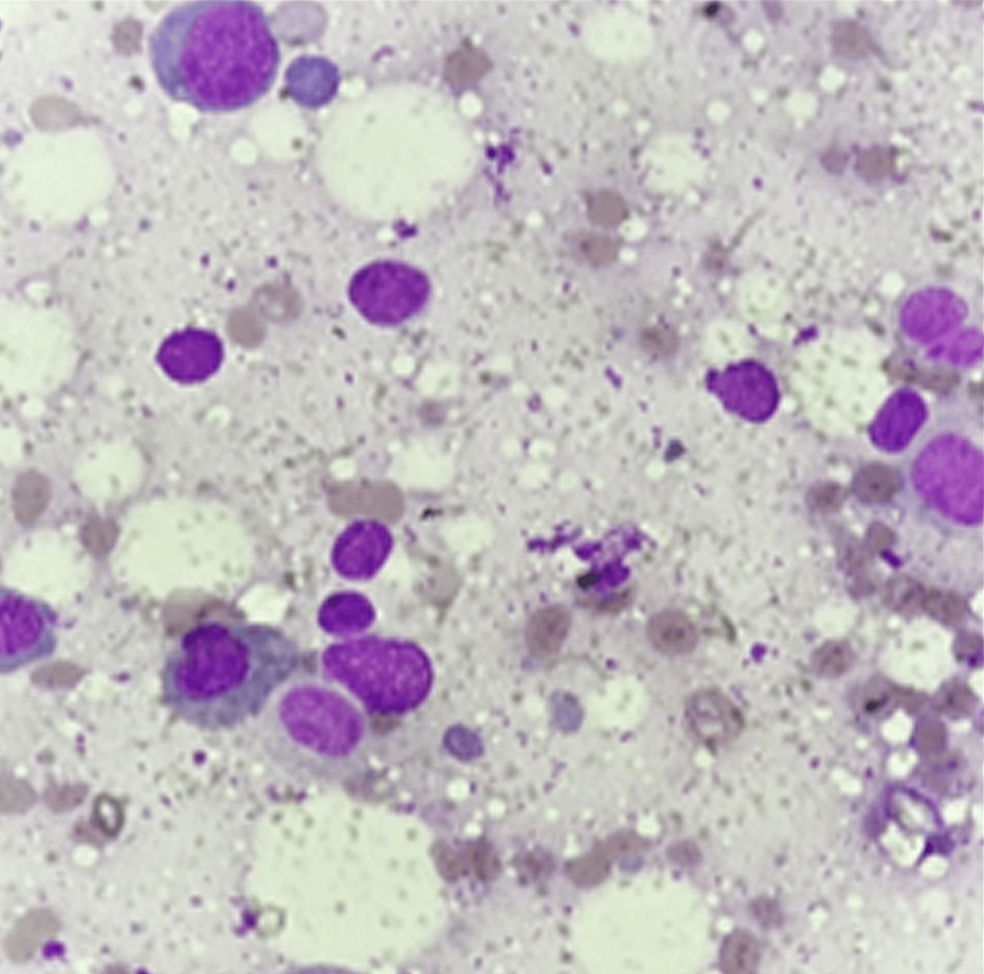
Bone marrow aspiration, Giemsa stain, 400x magnification Bone marrow aspiration shows dyserythropoietic features including multinucleation

**Figure 6:**
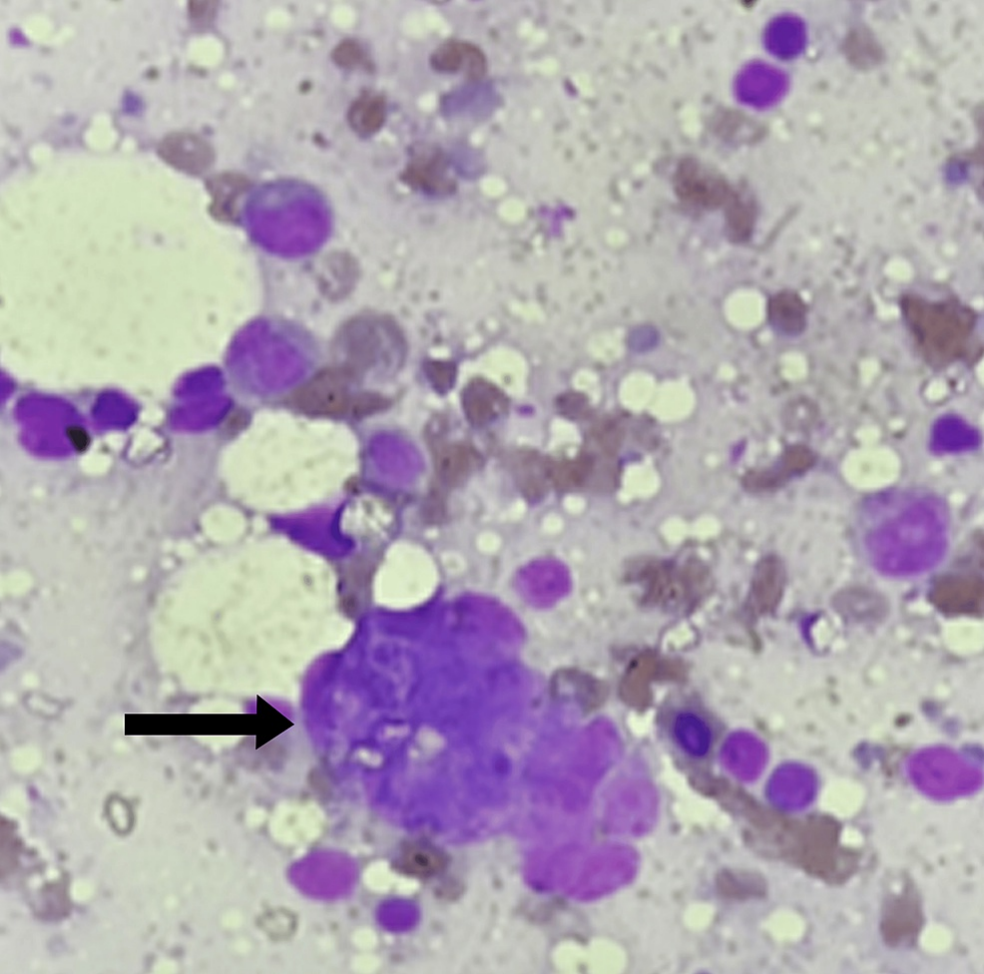
Bone marrow aspiration, Giemsa stain, 400x magnification Dyserythropoietic features including multinucleation and budding of a nucleus are denoted by a black arrow

It was reported as CDA-I which showed normocellular marrow with dyserythropoietic features including multinucleation and budding of the nucleus (Figures 5, 6). The mother reported that her antenatal period was unremarkable. The genetic documentation pertaining to the patient's familial background was not available.

## Discussion

CDA refers to a group of inherited disorders characterized by anomalies in the latter stages of erythropoiesis, leading to inefficient erythropoiesis and the subsequent development of hemochromatosis. Four major forms and several minor subgroups of CDA are identified. The consideration of CDA should be included in the differential diagnosis when there is insufficient reticulocytosis in terms of the severity of the anemia, along with unexplained hyperbilirubinemia and iron overload. CDA-I and CDA-II exhibit autosomal recessive inheritance, while types III and IV demonstrate autosomal dominant inheritance. The annual occurrence of CDA-I is reported to be 1 per 100,000 newborns [6]. To date, a total of over 300 cases have been documented globally [6]. CDA-I is a clinical condition that is infrequently observed and has primarily been documented in Central and Western European countries, as well as North Africa. There have been only a limited number of reported instances from the Indian subcontinent [7]. The autosomal recessive condition is usually caused by a change in the CDAN1 gene on chromosome 15, and this change is usually found in kids or teens [6]. The inheritance pattern for CDA-I is autosomal recessive. Each sibling of an afflicted person has a 25% risk of being affected, a 50% chance of being an asymptomatic carrier, and a 25% chance of being unaffected and not a carrier at conception if both parents are known to be heterozygous for a pathogenic variation that causes CDA-I [3].

The clinical presentations include a variety of symptoms, including anemia, jaundice, hepatomegaly, or splenomegaly. Extramedullary hematopoiesis in the paravertebral regions of the frontal and parietal bones of the skull may arise as a result of dyserythropoiesis. Dysmorphic characteristics such as syndactyly, supernumerary toes, or nail absence may manifest in around 4-14% of individuals diagnosed with CDA-I. In CDA-I, laboratory results often reveal a decrease in hemoglobin levels accompanied by insufficient reticulocyte counts related to the severity of the anemia. The bone marrow aspirates show erythroid hyperplasia, characterized by the presence of multiple nuclei and basophilic stippling, as well as the presence of polychromatic erythroblasts. The presence of incompletely split cells exhibiting chromatin bridges is a distinctive and characteristic finding in CDA-I. The characteristic observation of red blood cell lysis in acidified serum of CDA-II is not present in CDA-I. A positive acidified serum test shows that the person has a condition called hereditary erythroblastic multinuclearity. Electron microscopy is often regarded as the gold standard for diagnosing CDA-I, as it allows for the finding of the peculiar Swiss cheese pattern of heterochromatin [6].

The differential diagnosis includes many conditions such as thalassemia, some hemoglobinopathies, hereditary sideroblastic anemia, myelodysplasia, megaloblastic anemia (mostly caused by deficiencies in vitamin B12 or folate), and other forms of CDA [1]. The patient presented significant macrocytosis accompanied by dyserythropoiesis, a condition that is typically not present in cases of thalassemia or hemoglobinopathy. The absence of ringed sideroblasts and the lack of a microcytic blood picture effectively exclude the possibility of sideroblastic anemia [5]. The absence of dysplastic characteristics in the granulocytic/megakaryocytic lineage is indicative of the exclusion of myelodysplastic syndrome. The exclusion of other potential causes of macrocytosis, such as deficiencies in vitamin B12 or folate, is based on a lack of loose and fine chromatin structures in erythroblastic nuclei, the absence of giant granulopoietic cells, and the hyperlobulation of megakaryocytes [6].

The acidified serum lysis test yielded normal results, excluding the possibility of CDA-II. The examination of peripheral blood smears during the initial presentation, as well as the analysis of the bone marrow aspirate, indicated the presence of internuclear chromatin bridges. Additionally, erythroid hyperplasia was observed, characterized by megaloblastoid changes and multinuclear cells. The absence of giant erythroblasts was noted, which effectively eliminates the possibility of CDA-III [1].

The results of this study provided support for a diagnosis of CDA-I. The primary approach to managing CDA-I is providing supportive care, which may include the administration of red blood cell transfusions. Interferon therapy has the potential to decrease the need for transfusions. In pediatric patients, the presence of gallstones can require cholecystectomy. The primary long-term complication of hemosiderosis can manifest in children who have not received blood transfusions. This issue arises from the increased absorption of iron in the intestines, along with the deposition of iron caused by inadequate erythropoiesis. Multiple phlebotomy procedures have been shown to give normal ferritin levels, and in cases where children's serum ferritin levels exceed 1000ng/dl, oral chelation therapy may be indicated [6]. It is possible to perform prenatal and preimplantation genetic testing as well as carrier screening for relatives who are at risk after pathogenic variations in an affected family member have been found [3].

## Conclusions

CDA should be considered as a possible diagnosis in pediatric patients who present with refractory anemia, hepatosplenomegaly, erythroid hyperplasia, and evidence of dyserythropoiesis during the examination of the bone marrow. The presence of hyperbilirubinemia and iron overload in the absence of an identifiable cause also gives rise to suspicion of CDAs. The identification of CDA-I can be established based on the distinctive features observed in peripheral blood smears and bone marrow examinations. Due to the hereditary nature of the condition, we also advise that siblings and other close relatives of a diagnosed patient be evaluated. This will allow for the earliest start of the necessary treatment for the affected patients, as well as the monitoring of hemoglobin and iron levels. Currently, it is imperative to do additional studies in order to have in-depth knowledge of the long-term progression of this disease.
